# Determining the Efficacy of Octreotide on Lymphorrhea Formation in Patients With Early Breast Carcinoma After Modified Radical Mastectomy: A Randomized Controlled Trial

**DOI:** 10.7759/cureus.93374

**Published:** 2025-09-27

**Authors:** Sumit Bhaskar, Pawan K Singh, Priyesh Shukla, Ashok K Verma, Mahendra Singh

**Affiliations:** 1 Department of General Surgery, Ganesh Shankar Vidyarthi Memorial Medical College, Kanpur, IND; 2 Department of Radiodiagnosis, Ganesh Shankar Vidyarthi Memorial Medical College, Kanpur, IND; 3 Department of Pathology, Ganesh Shankar Vidyarthi Memorial Medical College, Kanpur, IND

**Keywords:** axillary lymph node dissection, breast cancer, lymphatic drainage, lymphorrhea, modified radical mastectomy, octreotide, postoperative complications, seroma

## Abstract

Background: Postoperative lymphorrhea and seroma formation are frequent complications after modified radical mastectomy (MRM) that can prolong recovery. Octreotide, a somatostatin analog with broad anti-secretory effects, has shown promise in reducing lymphatic drainage in various surgical settings. We conducted a randomized trial to evaluate the efficacy of octreotide in reducing lymphorrhea and seroma formation in patients with early breast cancer (stages I, IIA, and IIB) undergoing MRM.

Methods: Eighty patients with early breast carcinoma (stages I, IIA, and IIB) scheduled for MRM were randomly assigned to two groups (1:1 ratio). The octreotide group (n = 40) received injection octreotide subcutaneously 100 μg three times daily for five days and standard care postoperatively, while the control group received standard care without octreotide. Primary outcomes were the total volume of lymphorrhea drained until drain removal and the duration of drainage. Secondary outcomes included the incidence of postoperative seroma after drain removal, wound infection, flap necrosis, and length of hospital stay. Statistical analysis was performed using chi-square tests for categorical variables (α = 0.05) and t-tests for continuous variables.

Results: Octreotide significantly reduced the total lymphorrhea drainage volume (227.8 ± 181.4 ml; median 172.5, IQR 78.8-365.0) compared to the control group (364.1 ± 221.0 ml; median 352.5, IQR 198.8-562.5; U = 1081.5, *p* = 0.0068). The average duration of drainage was also shorter in the octreotide group (3.3 ± 1.7 days; median 3, IQR 2-4) than in the control group (4.1 ± 1.7 days; median 4, IQR 3-5; U = 1026, *p* = 0.0272). Daily drain output was significantly lower with octreotide (65.1 ± 41.4 ml/day; median 66.5, IQR 30-95) than in the control group (85.6 ± 48.7 ml/day; median 90, IQR 48-115; U = 1004.5, *p* = 0.0494). Seroma formation occurred in 30% of the octreotide group versus 35% of the control group (χ² = 0.06, *p* = 0.81). Wound infection (7.5% vs. 10%; OR = 0.73, *p* = 1.00) and flap necrosis (5% vs. 10%; OR = 0.47, *p* = 0.68) were low and did not differ significantly between groups. The mean postoperative hospital stay was slightly shorter with octreotide (5.8 ± 2.4 days; median 5, IQR 4-7) than with control (6.6 ± 2.3 days; median 6, IQR 5-8), although the difference did not reach statistical significance (U = 998.5, *p* = 0.054). No adverse drug-related effects (such as nausea, bradycardia, or hyperglycemia) were observed following octreotide administration.

Conclusion: Adjuvant octreotide significantly reduced the volume of lymphorrhea following MRM, with quicker drain removal indicating its potential to decrease drainage output. Although the octreotide group showed a trend towards lower seroma incidence, the relationship was not statistically significant in our sample. Octreotide was well tolerated with no significant side effects. These findings suggest octreotide can be a safe adjunct to standard surgical care to reduce lymphatic drainage after MRM. Larger studies may further clarify its impact on seroma formation and recovery.

## Introduction

Breast cancer is the most commonly diagnosed malignancy in women and the leading cause of cancer-related death among women globally. Approximately 2.3 million women are diagnosed annually, with over 670,000 deaths reported by WHO in 2022 alone [1]. Early detection and advancements in treatment modalities have improved survival rates. Surgical management, such as Breast Conserving surgery with postoperative radiotherapy, is considered the preferred local treatment for the majority of patients with early breast cancer, but axillary lymph node dissection is required when the sentinel lymph node biopsy is positive. Modified radical mastectomy (MRM) can be considered in cases where Breast conservation surgery or radiotherapy is not possible for early-stage breast cancer [2].

MRM is frequently associated with postoperative complications, among which seroma formation is the most common, with reported incidences ranging from 15% to 90% [3]. Seroma is defined as the accumulation of serous fluid in the dead space created post-axillary lymph node dissection, varies depending on the surgical technique, patient comorbidities, and postoperative care practices [3]. While not immediately life-threatening, it is a serious and disabling complication that contributes significantly to patient morbidity. It can result in prolonged drainage, delayed wound healing, flap necrosis, infection, and discomfort [4-6]. In some cases, it also delays the initiation of adjuvant therapy, thereby impacting overall treatment outcomes [4,5]. Despite the use of closed suction drains, flap fixation, and compressive dressings, seroma formation remains a persistent issue.

The pathophysiology of seroma formation is not fully understood, but it is believed to result from a combination of surgical trauma, lymphatic disruption, and inflammatory exudate accumulation [3]. The management of seroma has traditionally been supportive, with no standardized pharmacologic intervention showing consistent benefit.

Octreotide, a somatostatin synthetic analog, has been explored as a pharmacological option to reduce incidence of seroma. It exerts its effect through potent anti-secretory and vasoconstrictive effects, inhibiting the secretion of gastrointestinal hormones. Its ability to reduce splanchnic blood flow and triglyceride absorption is hypothesized to decrease the production of lymphatic fluid [7] and lymphatic flow [8]. Octreotide has been seen to be effective in managing lymphorrhea following thoracic duct injury, pelvic lymph node dissection, and other major surgeries [9,10]. A few preliminary studies have shown promising results in breast cancer surgeries, indicating its potential to reduce lymphatic drainage and seroma formation postoperatively [11].

Given the need for effective seroma prevention and the promising indications from prior studies, we conducted a randomized controlled trial to assess the efficacy of octreotide in reducing postoperative lymphorrhea and seroma formation in patients of early breast cancer undergoing MRM. We also evaluated secondary outcomes such as wound complications and hospital stay, to determine whether octreotide could facilitate overall recovery.

## Materials and methods

This study was a parallel-group, open-label, randomized controlled trial conducted at a tertiary care academic centre (Department of General Surgery, GSVM Medical College, Kanpur, India). The trial was registered in the Clinical Trials Registry and Registration No. CTRI/2024/12/077834 was obtained. Ethical approval was obtained from the institutional ethics committee, and written informed consent was obtained from all patients. Patient enrolment took place from December 2024 to May 2025.

Eligible patients were adult women aged 25-80 years diagnosed with early-stage breast cancer (clinical stage Ia, Ib, or II) who were scheduled for MRM. Key exclusion criteria included metastatic disease, prior axillary surgery, neoadjuvant chemotherapy (which might affect wound healing), uncontrolled diabetes, or other comorbidities such as thyroid disorders, hepatic or renal impairment, cardiac conditions, pancreatitis that could influence fluid accumulation, and known hypersensitivity to somatostatin analogs.

**Figure 1:**
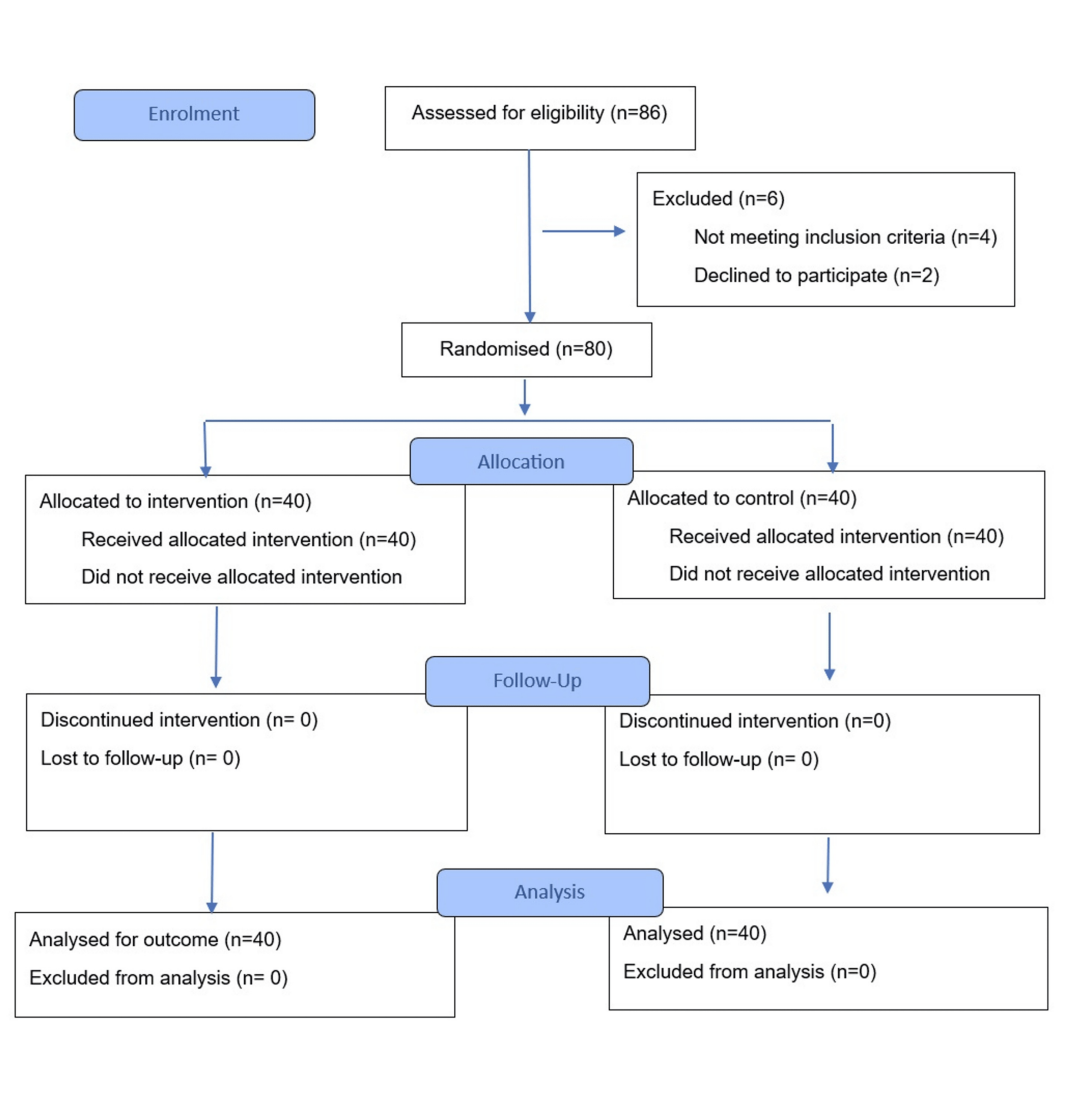
CONSORT diagram of this study. CONSORT: Consolidated Standards of Reporting Trials.

Eighty patients were included and randomly assigned in a 1:1 ratio to the octreotide group (intervention) or control group using simple randomization (computer-generated random numbers). All patients underwent a standard MRM under general anaesthesia following a uniform technique (axillary dissection, electrocautery for flap elevation, and placement of suction drains). In the octreotide group (n = 40), patients received octreotide 100 μg subcutaneously every eight hours (three times daily) starting on the first postoperative day and continuing for a total of five days. The control group (n = 40) received standard postoperative care without octreotide. Both groups otherwise received identical perioperative care, including antibiotics, analgesics, and wound management protocols. Suction drains (closed vacuum drains) were placed in the chest wall and axilla, and outputs were measured after 24 hours. Figure 1 represents the CONSORT flow diagram.

Drain output was measured daily for 10 postoperative days. Drains were removed after three consecutive days of <30 ml output. The primary outcomes included the total amount of lymphorrhea, calculated as the cumulative drainage volume in milliliters from the first postoperative day until drain removal, and the duration of drainage, measured as the number of days from surgery until the suction drain was taken out. These measures represent the overall lymphatic fluid production and the time until the patient no longer required a drain.

Secondary outcomes encompassed the average daily drainage volume, the total number of lymph nodes removed, and the number of positive nodes. Additional assessments included the occurrence of seroma, defined as the postoperative collection of clear serous fluid beneath the mastectomy flap, originating from disrupted lymphatic channels and small blood vessels during dissection, presented as a palpable fluid pocket or as serous leakage from the wound site, which was confirmed clinically and by aspiration if required; wound infection, identified within 30 days by redness, pus discharge, or a positive culture requiring antibiotic treatment; and flap necrosis, recorded as partial-thickness loss of the skin flap. The total length of hospital stay, counted in postoperative days from surgery to discharge, was also recorded.

Demographic and clinical data, such as age, number of lymph nodes excised, and the number of positive nodes, were collected for all participants. Drain output was measured every 24 hours starting 24 hours after surgery and continued until drain removal. Patients attended weekly follow-up visits for four weeks, followed by monthly visits for three months, to monitor seroma development and other complications.

For statistical evaluation, a sample size of 40 participants per group was calculated to achieve more than 80% power to detect a mean difference of 100 ml in drainage volume, assuming a standard deviation of approximately 200 ml, at a significance level of α = 0.05. Analyses were conducted using IBM SPSS Statistics for Windows, Version 25.0 (Released 2017; IBM Corp., Armonk, New York, United States). The continuous data were reported as mean ± standard deviation (SD) and median (interquartile range, IQR). Independent-samples t-test was applied for normally distributed data and Mann-Whitney U test for non-normally distributed data. Categorical data are presented as number of patients (n) and percentage (%). Between-group comparisons were performed using the chi-square test or Fisher’s exact test where appropriate. Reported statistics include chi-square (χ²) with degrees of freedom or odds ratio (OR) with corresponding p-values.

## Results

In this study, 86 patients were assessed for eligibility, of whom 80 patients met the inclusion criteria and were randomized (Figure 1). Six patients were excluded pre-randomization (four did not meet inclusion criteria and two patients declined participation). The two study groups (octreotide vs. control, 40 patients each) were similar in baseline characteristics (Table 1).

**Table 1:** Comparison of outcomes between the control and octreotide groups with p-value. Continuous data are presented as mean ± standard deviation (SD) and median (interquartile range, IQR). Independent-samples t-test was applied for normally distributed data and Mann–Whitney U test for non-normally distributed data. Reported statistics include the relevant test statistic (t or U) with *p*-values. Statistical significance was defined as *p* < 0.05.

Parameter	Control (Mean ± SD)	Octreotide (Mean ± SD)	Control Median (IQR)	Octreotide Median (IQR)	Test	Statistic	*p*-value	Significant
Age (years)	47.58 ± 9.84	47.23 ± 9.36	47.00 (39.00–56.00)	46.50 (39.00–56.50)	Mann–Whitney U	U = 809.00	0.9345	No
Operative time (minutes)	137.53 ± 18.85	135.03 ± 18.23	136.00 (127.00–145.00)	133.00 (123.25–144.50)	Independent t-test	t = 0.60	0.5482	No
Volume of lymphorrhea (ml)	364.12 ± 220.98	227.75 ± 181.36	352.50 (198.75–562.50)	172.50 (78.75–365.00)	Mann–Whitney U	U = 1081.50	0.0068	Yes
Duration of lymphorrhea (days)	4.05 ± 1.71	3.30 ± 1.71	4.00 (3.00–5.00)	3.00 (2.00–4.00)	Mann–Whitney U	U = 1026.00	0.0272	Yes
Duration of hospital stay (days)	6.61 ± 2.28	5.75 ± 2.38	6.00 (5.00–8.25)	5.00 (4.00–7.00)	Mann–Whitney U	U = 998.50	0.0541	No
Daily drain output (ml/day)	85.58 ± 48.69	65.08 ± 41.35	90.00 (47.50–115.00)	66.50 (30.00–95.00)	Mann–Whitney U	U = 1004.50	0.0494	Yes
Total lymph nodes dissected	13.30 ± 3.35	13.28 ± 3.15	12.00 (10.00–17.00)	12.00 (10.75–16.25)	Mann–Whitney U	U = 770.50	0.7755	No
Amount of lymphorrhea per lymph node (ml)	29.16 ± 19.67	18.19 ± 15.34	27.60 (15.96–41.38)	12.92 (6.04–29.33)	Mann–Whitney U	U = 1052.00	0.0155	Yes
Number of positive lymph nodes	1.27 ± 1.26	1.30 ± 1.42	1.00 (0.00–2.00)	1.00 (0.00–2.00)	Mann–Whitney U	U = 818.50	0.8567	No
Amount of lymphorrhea by positive lymph nodes (ml)	34.61 ± 42.77	17.77 ± 29.99	17.78 (0.00–61.31)	7.83 (0.00–22.47)	Mann–Whitney U	U = 945.00	0.1571	No

**Figure 2:**
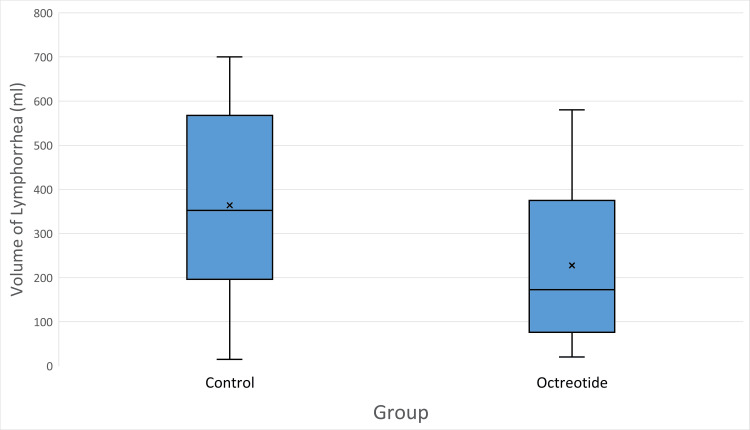
Comparison of volume of lymphorrhea (ml) between control and octreotide groups. The boxplots show the median (horizontal line), interquartile range (box), minimum and maximum values (whiskers), and mean (×). The octreotide group demonstrated a lower total lymphorrhea volume compared to the control group.

**Figure 3:**
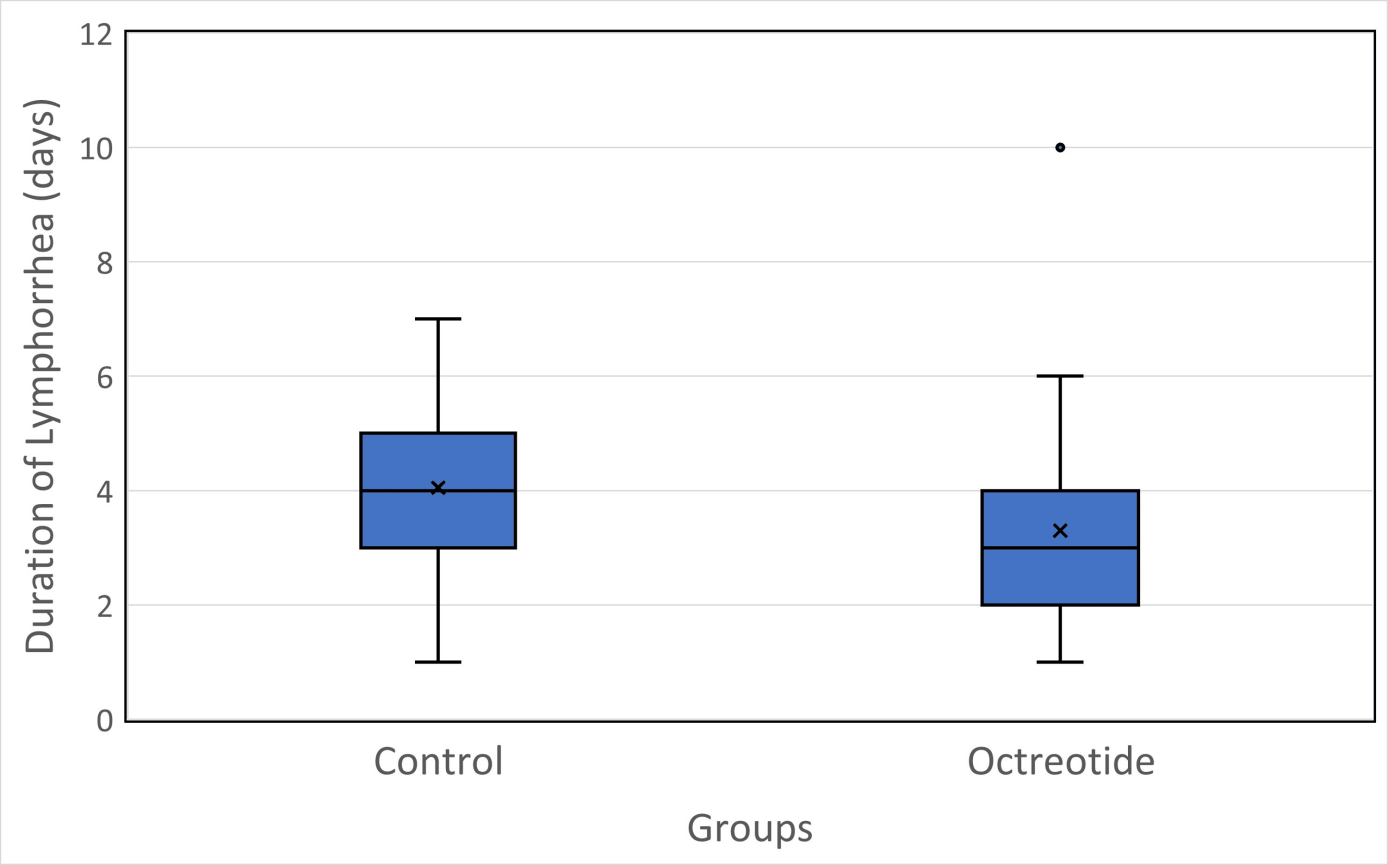
Comparison of duration of lymphorrhea (days) between control and octreotide groups. Boxplots depict the median (horizontal line), interquartile range (box), minimum and maximum values (whiskers), mean (×), and outliers (•). The octreotide group showed a shorter duration of lymphorrhea compared with the control group.

The primary outcome, total lymphorrhea volume accumulated in the drain, was significantly reduced in patients receiving octreotide. A substantial reduction of approximately 37.5% in the total mean postoperative volume of lymphorrhea (ml) was observed, and the total volume of lymphorrhea in the control group was 364.12 ± 220.98 ml, compared with 227.75 ± 181.36 ml in the octreotide group (U = 1081.5, *p-*value = 0.0068), as shown in Figure 2. The duration of lymphorrhea in the octreotide group (3.30 ± 1.71 days) was shorter than in the control group (4.05 ± 1.71 days). This difference was not statistically conclusive (U = 1026, *p-*value = 0.0272), but the trend suggested a faster cessation of lymphatic drainage in the treatment arm (as shown in Figure 3), with one notable outlier at 10 days (indicating a single octreotide patient who had unusually prolonged drainage). Overall, the octreotide group’s box is slightly lower on the y-axis, and its upper whisker is shorter, reflecting generally shorter lymphorrhea duration compared to the control.

**Figure 4:**
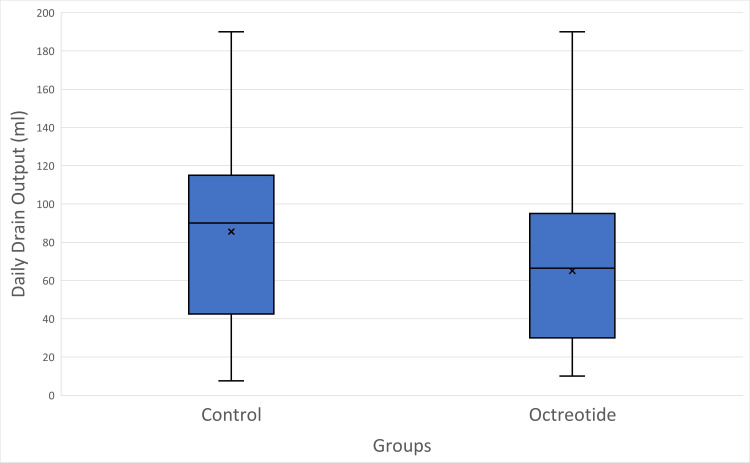
Comparison of daily drain output (ml) between control and octreotide groups. Boxplots illustrate the median (horizontal line), interquartile range (box), minimum and maximum values (whiskers), and mean (×). The octreotide group demonstrated a lower mean daily drain output compared with the control group.

The daily drain output was significantly lower in the octreotide group (65.08 ± 41.35 ml/day; median 66.5, IQR 30-95) compared with controls (85.58 ± 48.69 ml/day; median 90, IQR 47.5-115.0) (U = 1004.5, *p* = 0.0494), as shown in Figure 4, probably indicating reduced ongoing lymphatic leakage.

The duration of postoperative hospital stay was slightly reduced in the octreotide group, consistent with the slightly faster drain removal. Control patients had 6.61 ± 2.28 days (median 6, IQR 5-8) compared to 5.75 ± 2.38 days (median 5, IQR 4-7) in the octreotide group; however, this difference was not statistically significant (U = 998.5, *p* value = 0.054). Notably, 30% of octreotide patients were discharged by postoperative day 5 (once drains were out and criteria met) compared to 20% of controls, reflecting a trend toward earlier discharge in the intervention group.

**Figure 5:**
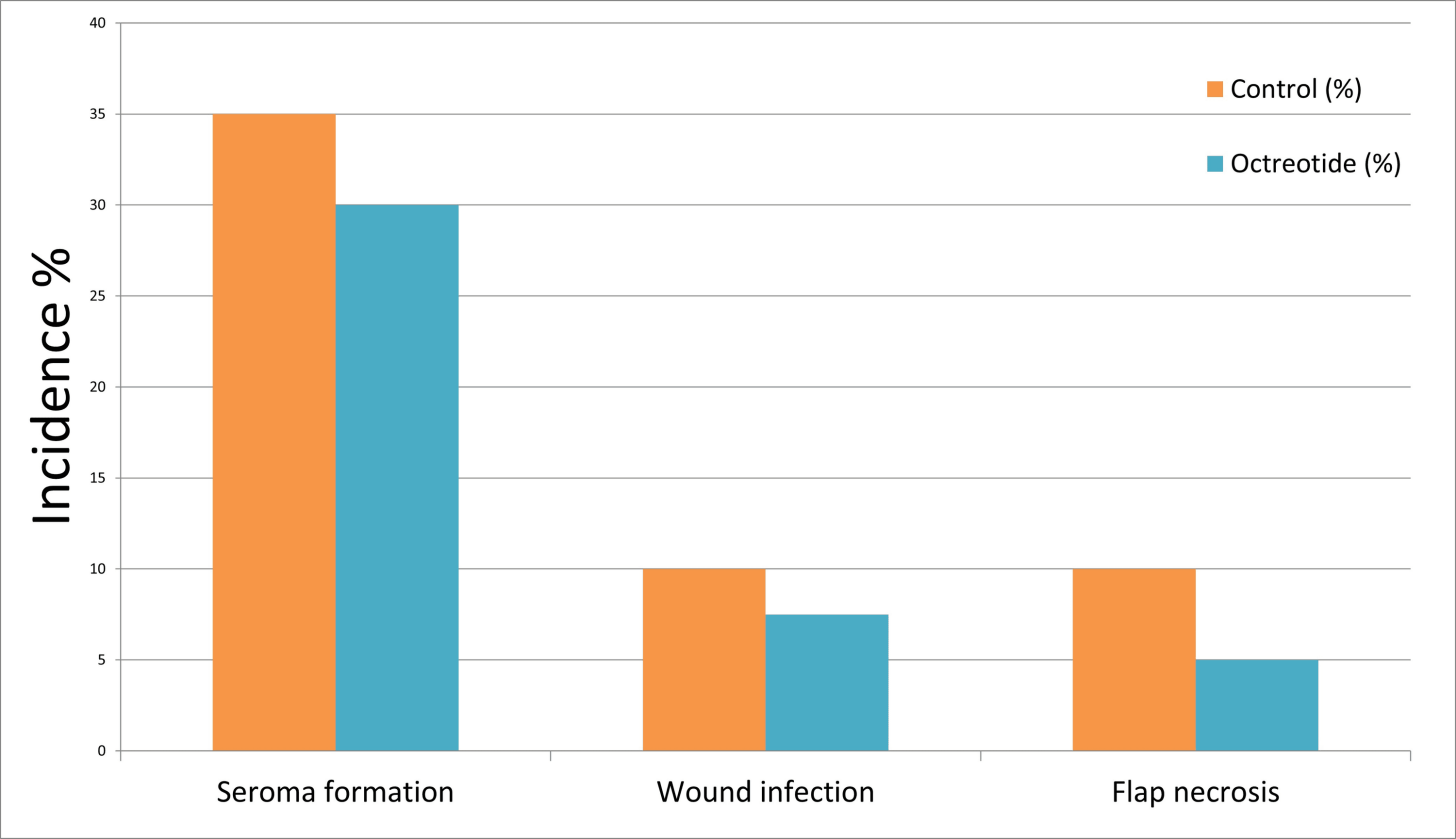
Postoperative complication rates in the control versus octreotide groups. Comparison of postoperative complication rates, including seroma formation, wound infection, and flap necrosis, between the control and octreotide groups. Values are expressed as percentages. The octreotide group demonstrated lower rates across all complications compared to the control group.

The extent of axillary dissection was similar between the two groups, with a mean of lymph nodes dissected in the control group (13.30 ± 3.35) and in the octreotide group (13.28 ± 3.15). When lymphorrhea volume was normalized to the number of lymph nodes dissected, the octreotide group value demonstrated markedly lower output (18.19 ± 15.34 ml/node; median 12.92, IQR 6.04-29.33) than the control group value (29.16 ± 19.67 ml/node; median 27.6, IQR 16-41) (U = 1052, *p* = 0.0155). Lymphorrhea per positive lymph node was significantly reduced with octreotide group (17.77 ± 29.99 ml; median 7.83, IQR 0-22.47) compared to controls (34.61 ± 42.77 ml; median 17.8, IQR 0-61.31, U = 945, *p* value = 0.157). These findings suggest that for a given surgical dissection extent or tumor burden, octreotide was associated with substantially less lymphatic drainage postoperatively, but the difference did not reach statistical significance. Postoperative wound complications were observed less frequently in the octreotide arm, as shown in Table 2 and Figure 5.

**Table 2:** Comparison of outcomes between the control and octreotide groups. Categorical data are presented as number of patients (n) and percentage (%). Between-group comparisons were performed using the chi-square test or Fisher’s exact test where appropriate. Reported statistics include chi-square (χ²) with degrees of freedom or OR with corresponding p-values. Statistical significance was defined as *p* < 0.05. OR: odds ratio.

Variable	Control group (n = 40)	Octreotide group (n = 40)	Test	Statistic value	*p*-value	Significant
Wound infection (%)	4 (10.0%)	3 (7.5%)	Fisher’s Exact	OR = 0.73	1.0000	No
Flap necrosis(%)	4 (10.0%)	2 (5.0%)	Fisher’s Exact	OR = 0.47	0.6752	No
Seroma formation on follow-up (%)	14 (35.0%)	12 (30.0%)	Chi-square	χ² = 0.06, df=1	0.8113	No

Despite the reduced drainage volumes, the incidence of seroma (fluid re-accumulation after drain removal) was not significantly different between groups. In the control group, 14 out of 40 patients (35%) developed a palpable seroma requiring needle aspiration (if more than 50 ml). In the octreotide group, 12 out of 40 patients (30%) experienced seroma formation. The difference (35% vs. 30%) was not statistically significant (χ² = 0.06, *p* value = 0.81). Most seromas were small and occurred within the first two weeks after drain removal. All resolved after one or two aspirations with no further complications.

Wound infections were seen in four patients (10%) in the control group value and three patients (7.5%) in the octreotide group. These infections were superficial and managed successfully with antibiotics and dressings; no patient required re-operation for infection. There was no significant difference in infection rate between groups (OR = 0.73, *p* value = 1.00 ). Flap necrosis was observed in four control patients (10%) versus two octreotide patients (5%), which was not statistically significant (OR = 0.47, *p* = 0.68). None of these differences were statistically significant. All instances of necrosis were minor (small areas of the skin flap edge) and managed with conservative management. Figure 5 illustrates these postoperative morbidity outcomes.

Overall, the results demonstrate that subcutaneous octreotide injections led to a meaningful reduction in lymphatic fluid drainage after MRM, without increasing complications. The primary endpoints (total volume of lymphorrhea and daily output) were significantly improved with octreotide, and several secondary measures trended toward better outcomes in the treatment group.

Adverse effects

Importantly, no adverse effects attributable to octreotide were observed. There were no episodes of any significant gastrointestinal symptoms, symptomatic bradycardia, or hyperglycaemia necessitating intervention. Octreotide injections were well tolerated, and no patients required discontinuation of the drug.

In summary, the administration of octreotide in the postoperative period significantly reduced lymphatic drainage volume and showed a trend toward faster drain removal. However, the reduction in seroma by using octreotide is not significant in our study. Postoperative complications were uncommon in both groups and were not worsened by octreotide. Complication rates of MRM, such as flap necrosis and wound infection, were lower in the octreotide group, although not statistically significant.

## Discussion

In this randomized trial, octreotide used after MRM markedly reduced postoperative lymphorrhea drainage volume, which was 37% reduced in the octreotide group, and reduced daily drain out, which was statistically significant, whereas shorter duration of lymphorrhea, hospital stay, and lower seroma incidence with octreotide compared to controls but not statistically significant. Similarly, Prajapati et al. found that postoperative octreotide significantly shortened drainage time and hospital stay in mastectomy patients [11]. Carcoforo et al.’s large RCT also showed a significant reduction in seroma formation and wound infections with perioperative octreotide [12]. In pelvic surgery, Kim et al. observed that octreotide dramatically reduced daily lymph output, shortened drain duration, and decreased hospitalization after radical prostatectomy lymphadenectomy [13], mirroring our effects on lymphorrhea. Likewise, in kidney transplant patients with lymphatic leaks, Baderiya et al. reported that octreotide shortened lymphorrhea duration (11.6 vs. 16.2 days) relative to conservative management [14]. These consistent findings across diverse surgeries (breast, pelvic, transplant) support the biological rationale that somatostatin analogs suppress lymphatic flow by splanchnic vasoconstriction and anti-inflammatory effects.

Chéreau et al., in a phase 2 trial of the somatostatin analog, pasireotide, showed only a non-significant trend toward reduced lymphocele rates after axillary dissection [15]. These discrepancies may reflect differences in drug type (octreotide vs. pasireotide), dosing schedules, timing (perioperative vs. delayed administration), and patient/surgical factors. For example, Ayandipo et al. used intravenous octreotide 100 µg every eight hours for five days after mastectomy and found non-significant trends (11 vs. 13 days to drain removal) [16], suggesting their study may have been underpowered to detect moderate effects. In contrast, most positive trials (including ours) used subcutaneous octreotide 100 µg three times daily. Surgical technique and risk factors may also differ. Nagayama et al. reviewed that extensive axillary dissection and patient factors (obesity, hypertension) strongly influence seroma risk, which could mask drug effects [7].

Systematic reviews to date conclude that evidence for somatostatin analogs in seroma reduction is limited. A recent meta-analysis by Hirono et al. found only a slight decrease in drained volume with somatostatin analogs and no significant change in drainage duration or seroma incidence [17]. Our results contribute to this mixed picture: we observed clear benefits in drainage time and drain volume reduction, but post drain removal seroma formation did not reach significance. Discrepancies across studies may hinge on sample size, statistical power, and endpoint definitions.

In summary, our findings align with several trials indicating that postoperative octreotide can safely reduce postoperative lymphatic drainage after nodal dissection. However, other RCTs have failed to show significant effects, underscoring the need to define which patients benefit most. Heterogeneity in surgery types (breast vs. pelvic), somatostatin analog used, and drainage protocols likely explains much of the variability. Future large, multicentre trials with standardized protocols are needed to clarify the role of octreotide in seroma prevention. Meanwhile, given its low risk profile, octreotide could be considered in high-risk breast surgery cases (extensive axillary clearance or patients with comorbidities) while recognizing that routine use is not yet universally endorsed.

## Conclusions

This study indicates that octreotide can play a beneficial role in effectively reducing postoperative lymphorrhea drainage volume and drainage duration in patients undergoing MRM for early breast cancer. The overall outcomes suggest that octreotide contributes to a more favorable postoperative course without increasing complications such as wound infection or flap necrosis. Taken together, these findings highlight octreotide as a safe and potentially useful adjunct in surgical management to ease recovery and improve patient comfort, warranting further exploration in larger and longer-term studies to better define its role in routine clinical practice.
